# No effect of omeprazole on pH of exhaled breath condensate in cough associated with gastro-oesophageal reflux

**DOI:** 10.1186/1745-9974-1-10

**Published:** 2005-10-19

**Authors:** Alfonso Torrego, Stefan Cimbollek, Mark Hew, Kian Fan Chung

**Affiliations:** 1 Department of Thoracic Medicine, National Heart & Lung Institute, Imperial College and Royal Brompton Hospital, London, UK

## Abstract

**Background:**

Endogenous airway acidification evaluated as pH in exhaled breath condensate (EBC) has been described in patients with chronic cough. Proton pump inhibitors improve gastro-oesophageal reflux (GOR)-associated cough.

**Methods:**

We examined pH levels in EBC and capsaicin cough response in 13 patients with chronic cough (mean age 41 years, SD 9) associated with GOR before and after omeprazole treatment (40 mg/day for 14 days) and its relationship with clinical response.

**Results:**

Omeprazole abolished symptoms associated with GOR. Patients with chronic cough had an EBC pH of 8.28 (SD 0.13) prior to treatment but this did not change with omeprazole treatment. There was a significant improvement in the Leicester Cough Questionnaire symptom scores from 80.8 points (SD 13.2) to 95.1 (SD 17) (p = 0.02) and in a 6-point scale of cough scores, but there was no change in capsaicin cough response.

**Conclusion:**

An improvement in GOR-associated cough was not associated with changes in EBC pH or capsaicin cough response. These parameters are not useful markers of therapeutic response.

## Introduction

Chronic cough, conventionally defined as a cough persisting for more than 8 weeks, is a common respiratory problem and, at times, presents as a difficult management issue. Asthma, rhino-sinusitis and gastro-oesophageal reflux (GOR) have been identified as the most common diagnoses associated with chronic cough [1]. GOR alone or in combination with other factors is the cause of chronic cough in 10–40% of adult patients [2,3]. Two main pathogenic mechanisms in GOR related cough have been described: micro-aspiration of gastric contents and a vagally-mediated oesophageal-tracheobronchial reflex [4]. The acid content of the refluxate may be an important component of the cough trigger associated with GOR, and this is supported by the fact that the chronic cough in some patients associated with GOR is improved or controlled by proton pump inhibitors that suppress gastric acid output [3,5,6]. Therefore, reflux of the gastric acid could directly activate cough receptors in the upper airways or indirectly through an oesophageal-tracheobronchial reflex [7].

Exhaled breath condensate (EBC) is a simple non-invasive technique for the monitoring of airway inflammation, since it may be representative of the epithelial lining fluid. Endogenous airway acidification, as assessed by the pH of exhaled breath condensate, has been reported in patients with non-asthmatic chronic cough, including GOR [8]. The fall in pH represented a doubling in the amount of H^+ ^ions and this could contribute to the sensitised cough reflex measured with capsaicin since an acid environment has been shown to activate Aδ and C fibres in the airways of rodents [7,9].

In order to examine further the significance of acid pH in the pathogenesis of GOR-associated cough, we measured pH of exhaled breath condensate in patients with chronic cough associated with abnormal lower oesophageal pH. We determined whether the improvement in cough associated with treatment with proton pump inhibitors was associated with changes in capsaicin responsiveness and in EBC pH.

## Methods

### Subjects

We recruited 13 patients with chronic cough (age 41 ± 9, 5 males) defined as a cough persisting for more than 2 months, associated solely with GOR as defined by an abnormal 24-hour oesophageal pH measurement from our Cough Clinic. In these patients, we had excluded the presence of asthma and rhino-sinusitis. FEV_1 _(predicted value: 99.8 ± 8.0%) and FVC (103 ± 8.0%) were within the normal range. The chest radiograph was normal and histamine responsiveness measured as PC_20 _(the concentration of histamine causing a 20% fall in FEV_1_) as greater than 16 mg/ml. Skin prick to common allergens were negative and they had no nasal symptoms. Eight of 13 patients reported symptoms of heartburn, regurgitation or dyspepsia; the rest were asymptomatic. All participants were non-smokers. All subjects gave informed consent to participate in the study which was approved by the Royal Brompton and Harefield NHS Trust Ethics Committee.

### Oesophageal pH study

An ambulatory 24 hour pH study was performed with the Synectics Digitrapper Mk III (Synectics Medical A/B, Sweden). An Antimony pH electrode was placed just above the upper border of the lower oesophageal sphincter. An acid reflux episode was defined as a drop in pH below 4.0. Significant reflux was defined as the total duration of reflux episodes exceeding 3.4% of the total study time.

### Symptom questionnaire

Cough severity was assessed using the Leicester Cough Questionnaire [10]. This consist of 19 questions (scored from 1 to 7 points each) relating to quality of life issues associated with chronic cough. A higher score indicates better health status and the range of the scale is from 19 to 133. Additionally, we used a 6-scale incremental cough symptom score with 0 as being no cough and 5 being the worst score for distressing cough most of the time [11].

### Capsaicin cough challenge

Capsaicin (8-methyl-N-vanillyl-6-nonenamide, 98%) obtained from Sigma-Aldrich, Gillingham, UK, was dispensed from a nebuliser chamber attached to a breath-activated dosimeter (PK Morgan Ltd, Gillingham, Kent, UK) set at driving pressure of 22 lbs/sq inch and a dosing period of 1 second. As described previously by Lalloo [12], the procedure started with the inhalation of 0.9% sodium chloride, followed with doubling doses of capsaicin from 0.976 μM (dose number 1) until 500 μM (dose number 10). The test was terminated when the subject coughed 5 times or more. The concentration of capsaicin causing 5 coughs or more (C5) was recorded.

### Exhaled breath condensate collection

Exhaled breath condensate (EBC) was obtained non-invasively by using a condenser (EcoScreen; Jaeger; Wurzburg, Germany) that collected the nongaseous components of the expiratory air. Subjects breathed tidally through a mouthpiece and a two-way non-rebreathing valve, which also served as a saliva trap. They were asked to breathe at a normal frequency and tidal volume, wearing a nose clip, for a period of 10 min. If subjects felt saliva in their mouth, they were instructed to swallow it. The condensate (at least 1 ml) was collected on ice at -20°C, and was transferred to 15 ml Corning tubes. Measurement of pH was performed following de-aeration with argon (350 ml/min for 10 min), using a pH meter (Jenway 350 pH meter, Spectronic Instruments, Leeds, UK).

### Study design

EBC collection, spirometry and capsaicin challenge were performed on the same day in this order. These measurements were performed before and after treatment with omeprazole (40 mg/day for 14 days)

### Statistical analysis

Data were analysed using Graph-Prism version 3.0 (Graph-Pad Software, San Diego, CA, US). Data are expressed as the mean ± SD. Differences between groups were determined using the Mann-Whitney U test. Capsaicin C5 values were analysed as log_10_C5. All reported p values are two-tailed. A p value of less than 0.05 was considered statistically significant.

## Results

**Figure 1:**
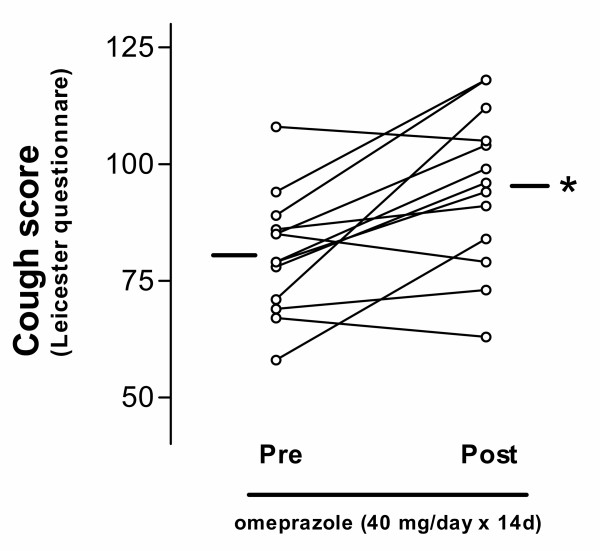
Cough scores measured by the Leicester Cough Questionnaire before and after 2 weeks of omeprazole treatment. * p = 0.02.

**Figure 2:**
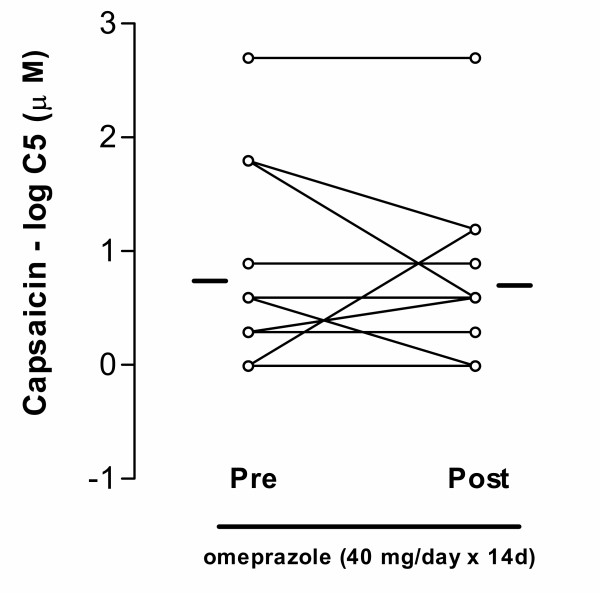
Cough reflex sensitivity to inhaled capsaicin measured as the concentration of capsaicin causing 5 or more coughs (C5) before and after omeprazole treatment.

**Figure 3:**
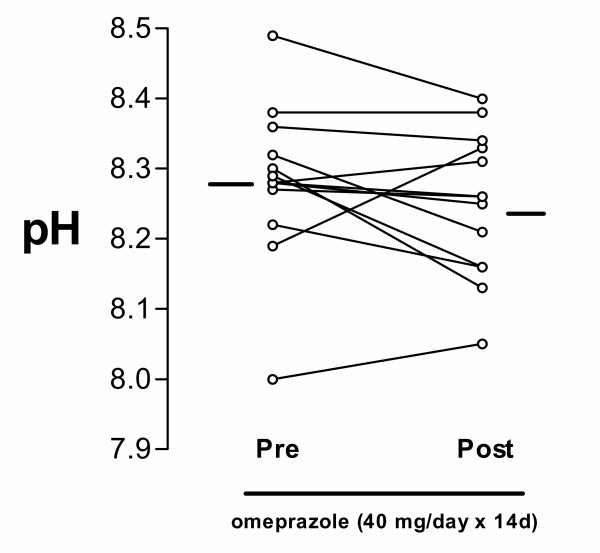
pH values in exhaled breath condensate in patients before and after omeprazole treatment. There was no effect of omeprazole.

The 8 patients with symptoms of gastro-oesophageal reflux reported disappearance of these symptoms. Using the Leicester cough questionnaire, in which the patients assessed their cough and related symptoms on a scale from 19 to 133 points, the patients reported a partial but significant symptomatic improvement after two weeks of omeprazole treatment (80.8 ± 13.2 vs. 95.1 ± 17 points, p = 0.02; Figure 1). Using the 6-point symptom score scale, we also found a reduction in cough score from 3.3 ± 0.7 to 2.6 ± 0.8 (p = 0.01). However, cough reflex sensitivity to capsaicin was not altered by omeprazole (log C5: 0.753 ± 0.23 vs. 0.707 ± 0.2; NS; Figure 2). There was no significant correlation between changes in the cough sensitivity reflex to inhaled capsaicin and the Leicester cough score. The log C5 was significantly lower than that measured in a cohort of 80 non-coughing normal volunteers (log C5: 1.83 ± 0.89; p < 0.0001), indicating that the coughers had a sensitised cough reflex. The pH of EBC was 8.28 ± 0.1 and did not change after 2 weeks of omeprazole treatment 8.25 ± 0.1 (Fig 3). EBC pH did not correlate with symptoms or with log C5.

## Discussion

After 2 weeks' treatment with omeprazole, we found a partial but significant clinical improvement in cough severity as assessed using the validated Leicester cough questionnaire. This was not accompanied by changes in capsaicin cough response or by changes in pH of the exhaled breath condensate. We conclude that these measurements do not reflect the clinical response. Additionally, omeprazole does not change the pH of exhaled breath condensate, most likely a reflection of the lack of change in pH of the epithelial lining fluid. This may also indicate that direct reflux of gastric acid into the upper airway is an unlikely explanation of GOR-associated cough.

GOR is a common associated cause of chronic cough and treatment with gastric acid suppressing proton pump inhibitors is often effective in controlling cough [3,5,6]. We ascertained the presence of GOR by performing 24-hour lower oesophageal pH monitoring in 13 patients, in whom only 8 had symptoms of GOR. Although the main purpose of the study was to determine any change in pH of the exhaled breath condensate, we did find a significant improvement in cough severity after 14 days of treatment. This indicates that the therapeutic response resulting from suppression of GOR by proton pump inhibitors occurs rapidly. In a recent open study by Poe and Kallay, improvement in cough was observed in 16 of 42 patients at 2 weeks and in 38 at 4 weeks [3]. Therefore, we might have seen further improvement with prolonged treatment. The short duration of treatment might be a limitation of our study.

The baseline EBC pH value in our patients was not lower than that previously published for healthy controls [13,14]. However, in a previous study performed in our department, Niimi et al [8] found that the mean EBC pH of patients with cough due to GOR was significantly lower (7.90) than in our present study. A possible explanation for this discrepancy may due to the small number of patients with GOR-associated cough included in Niimi's work (n = 5) and the fact that one of the patients had an uncharacteristically low pH. If this outlier were to be excluded, the other 4 values would be in a similar range to ours.

The pathophysiological mechanisms underlying GOR-associated cough are not fully understood. Micro-aspiration of oesophageal contents into the larynx and tracheobronchial tree is one of the possible explanations [15]. Our study indicates that this is unlikely since suppression of gastric acid by omeprazole did not alter the pH of exhaled breath condensate. Yorulmaz et al, in a recently published work, could not demonstrate a significant relationship between acid reflux episodes, pH variations in the upper oesophageal segments and symptoms of laryngeal irritation such as cough [16].

We found that cough sensitivity to capsaicin was increased when compared to a group of historical non-coughing normal volunteers [17,18]. However, there was no effect of omeprazole on capsaicin sensitivity, despite a significant symptomatic improvement in cough, a finding that has been previously reported [18]. In one report, where capsaicin cough reflex improved after omeprazole, the patients had more severe GOR symptoms including posterior laryngitis and acid flooding of the oesophagus [17].

In conclusion, our results indicate that EBC pH measurement is not a good tool in the follow-up of GOR-associated chronic cough during treatment with a proton pump inhibitors. In GOR, episodes of micro-aspiration are short and do not produce a persisting level of airway acidification, which may be the reason why changes in EBC pH are not detected.
